# The parameters of gait analysis related to ambulatory and balance functions in hemiplegic stroke patients: a gait analysis study

**DOI:** 10.1186/s12883-021-02072-4

**Published:** 2021-01-27

**Authors:** Min Cheol Chang, Byung Joo Lee, Na-Young Joo, Donghwi Park

**Affiliations:** 1 Department of Rehabilitation Medicine, College of Medicine, Yeungnam University, Daegu, Republic of Korea; 2 Department of Rehabilitation Medicine, Daegu Fatima Hospital, Daegu, Republic of Korea; 3 Department of Physical Medicine and Rehabilitation, Ulsan University Hospital, University of Ulsan College of Medicine, 877 Bangeojin sunhwando- ro, Dong-gu, 44033 Ulsan, Republic of Korea

**Keywords:** Gait analysis, Gait speed, Hemiplegia, Stroke, Gait disturbance

## Abstract

**Background:**

Ambulatory and balance functions are important for maintaining general health in humans. Gait analysis allows clinicians and researchers to identify the parameters to be focused on when assessing balance and ambulatory functions. In this study, we performed gait analysis with pressure sensors to identify the gait-analysis parameters related to balance and ambulatory functions in hemiplegic stroke patients.

**Methods:**

We retrospectively reviewed the medical records of 102 patients with hemiplegic stroke who underwent gait analysis. Correlations between various temporospatial parameters in the gait analysis and the motor and balance functions assessed using functional ambulation category, modified Barthel index, and Berg balance scale were analyzed.

**Results:**

Gait speed/height and the lower-limb stance-phase time/height were the only temporal and spatial parameters, respectively, that showed a statistical correlation with motor and balance functions.

**Conclusions:**

Measurements of walking speed and stance-phase time of the unaffected lower limb can allow clinicians to easily assess the ambulatory and balance functions of hemiplegic stroke patients. Rehabilitative treatment focusing on increasing gait speed and shortening the stance-phase time of the unaffected side may improve the ambulatory and balance functions in these patients.

## Background

Ambulatory and balance functions, which are basic functions necessary for independence in daily activities, are also important for mobility and maintenance of general health in humans [1]. Stroke, one of the most common causes of impaired ambulatory and balance functions [2], affects patients’ activities of daily living and ultimately limits their participation in community-based activities [3, 4]. Moreover, the stroke-induced impairments in ambulatory and balance functions frequently result in falls and fall-related injuries [5]. Thus, improvement of ambulatory and balance functions is one of the primary goals of stroke rehabilitation programs.

Gait analysis has been widely used in studies assessing ambulatory and balance function during gait.[2] Gait analyses can yield quantitative data for several temporospatial gait-related parameters, including gait speed, stride or step length, stance time, and the angle of each joint [6]. Knowledge of the elements related to ambulatory and balance functions can help clinicians identify the relevant factors during rehabilitative treatment of stroke patients and assist clinicians and researchers in determining the gait-related parameters that should be focused on when assessing balance and ambulatory functions. Gait dysfunctions in stroke patients are usually attributable to cognitive, motor, and sensory impairments [7–9], and may manifest as decreased walking speed, altered proportions of temporal phases, increased variability in the stride duration, and increased asymmetries in the single-stance phase [10, 11]. However, the multicollinearity of spatiotemporal data has made interpretation of spatiotemporal parameters difficult and led to uncertainty about the relevant gait-analysis parameters for assessment of poststroke gait. Moreover, although gait analysis can be used to simply measure the ambulatory and balance function of patients, the complexity and vastness of gait-analysis data precludes such an approach in actual clinical practice.

Therefore, in the current study, we used a gait-analysis tool with pressure sensors to investigate the parameters related to balance and ambulatory functions in hemiplegic stroke patients.

## Methods

### Participants

Our study was approved by the Ethics Committee (2020-07-020) and was conducted in accordance with the principles of the Declaration of Helsinki for human experiments. All methods followed the relevant guidelines and regulations. We retrospectively reviewed the medical records of patients with stroke who were admitted to our hospital between January 2017 and August 2020.

The inclusion criteria were as follows: (1) adult patients (age ≥ 20 years), (2) hemiplegia due to stroke, (3) ability to walk independently, (4) completed both gait analysis and clinical assessment of motor and gait functions, including modified Barthel index (MBI) evaluation, manual muscle test (MMT) of both lower extremities, Berg balance scale (BBS) measurements, and functional ambulation category (FAC) assessments. The exclusion criteria were as follows: (1) > 1-week interval between assessment of gait and motor functions and gait analysis, and (2) a history of other neurologic or musculoskeletal disorders that could affect the results of this study.

### Clinical assessment

Patients’ clinical records, including demographic data, clinical diagnosis, disease duration, Mini Mental Status Examination (MMSE) score,[12] MBI sub-scores for ambulation and stair-climbing [13], MMT score [14, 15], FAC [16], and BBS score [17], were evaluated. We also evaluated the spasticity of the hemi-side ankle plantar flexor muscle by using the modified Ashworth scale [18]. Using the MMT scores for both lower extremities, we calculated the total score (both hip flexor MMT + hip extensor MMT + knee extensor MMT + knee flexor MMT + ankle dorsiflexor MMT + ankle plantar flexor MMT; total score, 60), and the total score for the antigravity muscles in the lower extremities (both hip extensor MMT + knee extensor MMT + ankle plantar flexor MMT; total score, 30) [14, 15].

All clinical assessments were performed by the patient’s main physical therapist within 7 days of gait analysis. All therapists who performed the assessments were blinded to the results of gait analysis.

### Gait analysis

A computerized gait-analysis system (Walkway MG-1000; Anima, Japan) was used for gait analysis [19]. This system measures the temporospatial parameters of gait by analyzing on/off signals between the patient’s foot and the surface of the sensors at a sampling frequency of 100 Hz [19]. The length and width of the system’s walkway are 4.8 m and 0.82 m, respectively. During gait, data were obtained and processed using software embedded in the system [19].

### Experiment procedure

Participants wearing short pants were asked to get on a walkway and walk barefoot along a 12-m straight line, including 3.5 m in the front and 3.5 m beyond the end of walking path. Each participant performed one trial at a subjectively determined comfortable speed. A physical therapist with more than 10 years of experience performed gait analysis for all patients included in this study.

### Parameters of gait analysis

Measurements for the temporal parameters stance phase, swing phase, double stance phase, and stride were obtained in seconds. In addition, the duration of the stance phase during the total gait cycle, duration of the swing phase during the total gait cycle, and the duration of the double stance phase during the total gait cycle in both lower limbs were expressed as percentages. Measurements for the spatial parameters stride length, step length, and step width were obtained in centimeters. We also calculated the gait stability ratio (i.e., steps/m) for both lower limbs [20]. Additionally, gait angle (degrees), toe out angle (degrees), gait speed (cm/s), and cadence (step) of each lower limb were measured. The gait angle is the angle between the line of progression and the foot axis. It is zero when the foot axis is parallel to the line of progression, and positive when the foot axis points lateral to the line of progression. All measurement values were represented as mean ± standard deviation.

To compensate for variations in patient height, we calculated gait speed/height, gait speed/height^2^, stride length/height, stride length/height^2^, step length/height, step length/height^2^, step width/height, step width/height^2^, stride time/height, and stride time/height^2^.

### Statistical analysis

To identify the correlations between gait-analysis parameters and the results indicating motor function (FAC, ambulation sub-score of MBI, and BBS score), multiple linear regression analysis was used. To identify the variables affected by multicollinearity and the strengths of the correlations, testing for multicollinearity with variance inflation factors (VIFs) was performed. Multicollinearity was considered to be present when VIF was higher than 5 to 10. Multiple linear regression tests were performed after discarding parameters showing multicollinearity. Statistical analyses were performed using Statistical Package for the Social Sciences for Windows and R package for Windows (version 2.15.2; R Foundation for Statistical Computing, Vienna, Austria).

## Results

### Patient characteristics

A total of 102 patients with stroke (68 males and 34 females, 18–91 years of age, 148–185 cm in height, and 43–98 kg in weight) were investigated in this study. The results of the clinical assessments and gait analysis are presented in Tables 1 and 2. Mean height (cm), body weight (kg), disease duration (days), total MBI score, and MMSE were 165.04 ± 7.61 cm, 65.16 ± 11.92 kg, 29.20 ± 99.45 days, 80.36 ± 17.32, and 25.22 ± 6.11, respectively (Table 3). Among them, 93 patients had ischemic stroke lesions, while the remaining 9 had hemorrhagic stroke lesions. The average total MMT score for the lower-extremity muscles was 55.72 ± 4.36, while that for the antigravity muscles of both lower extremities was 27.44 ± 2.76.

**Table 1:** Clinical and demographic characteristics of included patients with hemiplegic stroke

	Patients
Age (years)	59.44±13.72
Sex (M:F)	68:34
Etiology (hemorrhagic stroke : infarction stroke)	9:93
Side (Left:Right)	43:59

*M:F *Male:female

**Table 2:** The temporo-spatial parameters of gait in patients with hemiplegic stroke

	Patients	
Height (cm)	165.04±7.61	
Weight (kg)	65.16±11.92	
Disease duration (days)	29.20±99.45	
MMSE	25.22±6.11	
Hemi-side ankle PF spasticity (MAS)	0.39±0.54	
Total BBS score	44.69±11.62	
Total MBI score	80.36±17.32	
MBI ambulation	10.86±5.14	
MBI stair climbing	3.31±4.19	
FAC	2.61±1.35	
Gait speed (cm/s)	48.62±16.75	
Cadence (step/min)	79.11±15.28	
Double stance time (s)	0.31±0.18	
	Affected side	Unaffected side
MMT hip flexor	4.35±0.56	5.0±0.0
MMT hip extensor	4.37±0.53	5.0±0.0
MMT Knee flexor	4.28±0.62	5.0±0.0
MMT knee extensor	4.29±0.61	5.0±0.0
MMT ankle DF	4.32±0.77	5.0±0.0
MMT ankle PF	4.33±0.75	5.0±0.0
MMT sum	25.96±3.56	25.0±0.0
Antigravity MMT sum	13.0±1.76	15.0±0.0
GSR	3.03±1.43	3.29±2.46
Stride length (cm)	72.37±17.85	72.28±19.39
Step length (cm)	36.38±9.34	36.17±10.39
Step width (cm)	12.67±4.61	12.87±4.40
Gait angle (°)	21.06±10.38	21.88±11.51
Toe angle (°)	9.38±7.79	10.50±6.90
Stance phase time (s)	2.23±11.48	1.13±0.40
Swing phase time (s)	0.47±0.13	0.42±0.17

*MBI* Modified Barthel's Index, *MAS* Modified Ashworth scale, *MMSE* Mini Mental Status Examination, *LE* Lower Extremity, *MMT* Manual Muscle Test, *sum* Summation, *GSR* Gait stability ratio, *DF* Dorsiflexor, *PF* Plantar flexor, *FAC* Functional ambulation classification, *BBS* Berg balance scale

**Table 3:** Multiple linear regression analysis for assessing temporo-spatial parameters of gait in predicting of balance and gait function

Dependent variable	Independent variables	R^2^	Beta coefficient	Standard error	Odd ratio (95% CI)	*P* value
BBS	Gait speed/height	0.227	0.476	0.130	0.308 ~ 0.826	<0.001
	Unaffected stance time/height	0.220	-0.470	7.832	-49.22~-17.93	<0.001
FAC	Gait speed/height	0.354	0.595	0.011	0.058 ~ 0.100	<0.001
	Unaffected stance time/height	0.199	-0.446	0.517	-3.597~-1.547	<0.001
Ambulation MBI	Gait speed/height	0.153	0.391	0.047	0.105 ~ 0.290	<0.001
	Unaffected stance time/height	0.136	-0.369	2.042	-12.153~-4.052	<0.001
Stair climbing MBI	Gait speed/height	0.181	0.426	0.037	0.101 ~ 0.249	<0.001
	Unaffected stance time/height	0.096	-0.310	1.700	-8.924~-2.178	0.001

*BBS* Berg Balance Scale, *FAC* Functional Ambulation Category, *MBI* Modified Barthel Index, *MMT* Manual Muscle Test, *Av* Average

### Correlation between Berg balance scale and gait‐analysis parameters

**Fig. 1:**
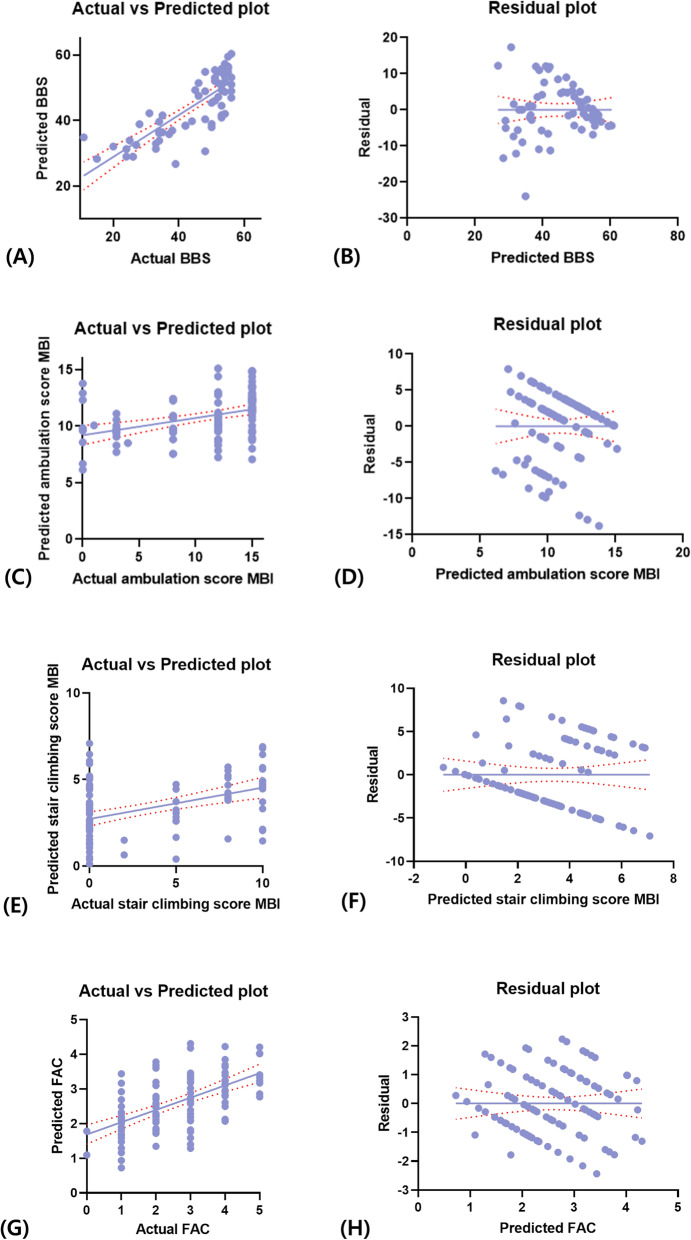
**a-b** Mean of the "actual vs. predicted plot" and the residual plot between the Berg balance scale (BBS) score and spatial parameters of gait (gait speed/height). **c-d** Mean of the "actual vs. predicted plot" and the residual plot between the ambulation sub-score of the modified Barthel index (MBI) and spatial parameters of gait (gait speed/height). **e-f** Mean of the "actual vs. predicted plot" and the residual plot between the stair-climbing sub-score of the MBI and spatial parameters of gait (gait speed/height). **g-h** Mean of the "actual vs predicted plot" and the residual plot between the functional ambulation category (FAC) and spatial parameters of gait (gait speed/height)

**Fig. 2:**
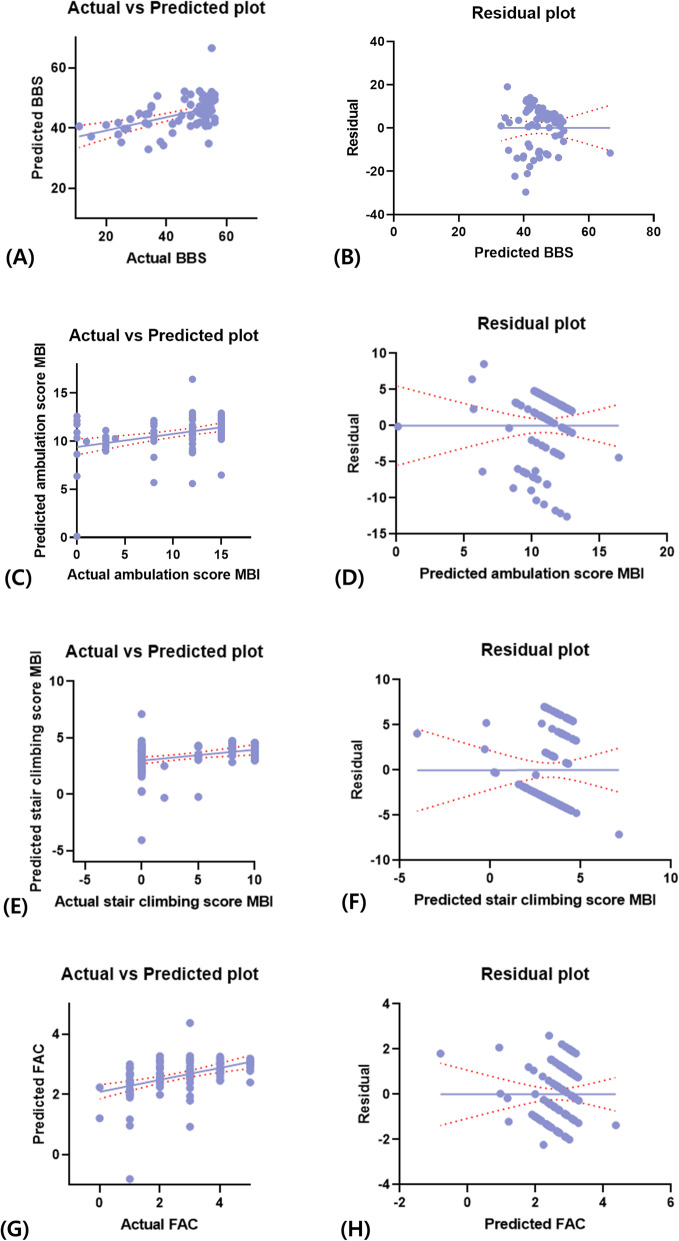
**a-b** Mean of the "actual vs. predicted plot" and the residual plot between theBerg balance scale (BBS) score and temporal parameters of gait (unaffected stance-phase time/height). **c-d** Mean of the "actual vs. predicted plot" and the residual plot between the ambulation sub-score of the modified Barthel index (MBI) and temporal parameters of gait (unaffected stance phase time/height). **e-f** Mean of the "actual vs. predicted plot" and the residual plot between the stair-climbing sub-score of the MBI and temporal parameters of gait (unaffected stance phase time/height). **g-h** Mean of the "actual vs. predicted plot" and the residual plot between the functional ambulation category (FAC) and temporal parameters of gait (unaffected stance phase time/height)

Among the spatial and temporal parameters of gait analysis, only gait speed/height and the unaffected-side stance-phase time/height, respectively, showed a statistically significant correlation with BBS scores (R^2^ = 0.227, p < 0.001; and R^2^ = 0.220, *p* < 0.001, respectively) (Figs. 1 and 2).

### Correlation between the ambulation sub-score of the modified Barthel index and gait-analysis parameters

Similar to the findings for BSS scores, gait speed/height and the unaffected-side stance-phase time/height were the only spatial and temporal parameters of gait analysis, respectively, that showed statistically significant correlations with the MBI ambulation sub-score (R^2^ = 0.153, *p* < 0.001; and R^2^ = 0.136, *p* < 0.001, respectively) (Figs. 1 and 2).

### Correlation between the stair climbing sub-score of modified Barthel index and gait-analysis parameters

In the analyses based on the stair climbing sub-score of MBI, gait speed/height and the unaffected-side stance-phase time/height were again the only spatial and temporal gait-analysis parameters, respectively, that showed statistically significant correlations with the stair climbing sub-score (R^2^ = 0.181, *p* < 0.001; and R^2^ = 0.096, *p* < 0.001, respectively) (Figs. 1 and 2).

### Correlation between functional ambulation category and gait‐analysis parameters

Consistent with the findings of the previous analyses, gait speed/height and the unaffected-side stance-phase time/height were the only spatial and temporal gait-analysis parameters, respectively, that showed statistically significant correlations with FAC (R^2^ = 0.354, *p* < 0.001; and R^2^ = 0.199, *p* < 0.001, respectively) (Figs. 1 and 2).

## Discussion

In this study, we analyzed various temporospatial parameters of gait analysis in patients with stroke and investigated the relationships between these parameters and parameters indicating ambulatory and balance functions. Our results showed that only gait speed/height and the unaffected-side lower-limb stance phase time/height were significantly correlated with the FAC and BBS and MBI scores.

Since gait speed is a reliable measure of ambulatory ability in patients with stroke, it is commonly used by clinicians and researchers [21]. Perry et al. [22] reported that gait speed differed significantly depending on the degree of impairment in ambulatory function: indoor-level ambulators (0.4 m/s), limited outdoor-level ambulators (0.40–0.80 m/s), and unlimited outdoor-level ambulators (> 0.8 m/s). Likewise, in our study, gait speed was significantly correlated with ambulatory and balance functions in all our patients. In addition to reflecting ambulatory and balance functions, gait speed evaluations offer the advantage of quick and easy measurements that can be performed without any equipment. Since the outcome of gait speed is related to mortality, poor quality of life, and physical and cognitive functional decline, clinicians and researchers can obtain information regarding the patient’s status and predict the functional prognosis by measuring gait speed [23, 24].

In our study, the unaffected-side stance-phase time was inversely correlated with the patients’ ambulatory and balance functions. A short stance-phase time of the lower limb on the unaffected side indicates a relatively longer swing-phase time [25]. In such patients, the lower limb on the affected side has to endure a longer stance phase, which is correlated with the limb’s motor function. However, the MMT scores for the affected side did not show correlations with any of the gait-analysis parameters. This finding can be interpreted as follows: Although muscle strength is an important factor influencing the support time of the lower limb on the affected side, other factors, such as proprioception or a subjective fear of falling, might also have a significant impact on ambulatory and balance functions [25, 26].

The results of our study can help identify the components that should be focused on during training to improve ambulatory and balance functions in hemiplegic stroke patients. To achieve this improvement, clinicians should formulate a strategy that enhances the walking speed and shortens the stance-phase time of the unaffected-side lower limb, thereby elongating the stance-phase time of the affected-side lower limb. Thus, a high walking speed and short stance-phase time of the unaffected-side lower limb can indicate good ambulatory and balance functions in hemiplegic stroke patients, and measurements of the walking speed and the stance-phase time of the unaffected-side lower limb can facilitate assessments of the ambulatory and balance functions in these patients. Rehabilitative treatment that focused on increasing the gait speed and shortening the stance-phase time of the unaffected side may help improve patients’ ambulatory and balance functions.

To our knowledge, this study is the first to evaluate the gait-analysis parameters related to ambulatory and balance functions in stroke patients. However, the study had some limitations. First, severely impaired patients were excluded from this study. Since the participants were requested to complete gait analysis, those with severe motor or balance impairment could not participate. Second, the patients underwent gait analysis without using any assistance, such as an ankle foot orthosis or a cane. Thus, if the patients’ gait function improved with such assistance, our results may not fully reflect the actual balance or gait functions. Future studies that address this limitation are warranted. Third, only the ambulation and stair-climbing sub-scores of MBI and the FAC were used in this study. However, for better evaluation, additional studies using various parameters that can adequately represent the patient’s ambulatory or balance function are needed. Fourth, we used only pressure sensor-based gait analysis without obtaining anthropometric measures. More accurate analysis of gait function would have been possible by using auxiliary data such as anthropometric measures along with pressure sensor-based measurements. However, our gait-analysis system based on pressure sensors allowed easy assessments, and the gait-analysis parameters assessed using pressure sensors adequately represented the ambulatory and balance functions of stroke patients with hemiplegia. The purpose of this study was to use pressure sensor-based gait analysis, which is easy to perform, to identify parameters that could more easily represent the patient’s ambulatory and balance function. However, more accurate data can be obtained if additional data such as anthropometric measures are used in future studies. Lastly, while the results showed significant correlations, the correlation coefficients were relatively low (r^2^ = 0.096 ~ 0.354), which may be attributed to the use of pressure sensor-based gait analysis. However, multiple previous studies using gait analysis provided insufficient explanation for this phenomenon. To overcome this limitation, further studies with larger patient populations and more varied biometric data may be necessary.

## Conclusions

Measurements of walking speed and stance-phase time of the unaffected lower limb can allow clinicians to easily assess the ambulatory and balance functions of hemiplegic stroke patients. Rehabilitative treatment focusing on increasing gait speed and shortening the stance-phase time of the unaffected side may improve the ambulatory and balance functions in these patients.

## Data Availability

The data sets in this study are available from the corresponding author on reasonable request.

## Abbreviations

MBI: Modified Barthel index (MBI)
MMT: Manual muscle test
BBS: Berg balance scale
FAC: Functional ambulation category
MMSE: Mini Mental Status Examination
VIFs: Variance inflation factors
